# Prothrombin time predicting time-dependent and risk-stratified mortality in polytrauma patients

**DOI:** 10.1186/s12245-025-00841-3

**Published:** 2025-04-02

**Authors:** Philipp Vetter, Cédric Niggli, Jan Hambrecht, Daniel Haschtmann, Hans-Christoph Pape, Ladislav Mica

**Affiliations:** 1 Department of Trauma Surgery, University Hospital Zurich, Zurich, 8091 Switzerland; 2 Department of Spine Surgery, Schulthess Clinic, Zurich, 8008 Switzerland

**Keywords:** Watson trauma pathway explorer, Trauma, Coagulopathy

## Abstract

**Background:**

Polytrauma is associated with a high mortality rate and often accompanied by coagulopathy. Prothrombin time (PT) is a prognostic factor for mortality in polytrauma patients.

The aim was to analyze the time- and severity-dependent role of PT in polytrauma patients related to mortality.

**Methods:**

Patients (≥ 16 years) with an Injury Severity Score ≥ 16 were retrospectively included, yielding 2890 cases after exclusion criteria. PT was measured at admission and 1, 2, 3, 4, 6, 8, 12, 24, and 48 h thereafter, reported as percentage activity of the reference reagence [%].

According to survival status, two groups were formed and compared. Binary logistic regression was used to test PT as an independent predictor for mortality. A closest top-left threshold method served for calculating threshold values between the survivor and non-survivor group. Patients were divided into subgroups according to PT levels and mortality was assessed for each subgroup at each time point.

**Results:**

PT values in the non-survivor group were lower throughout the measuring period (*p* < 0.05). PT threshold values declined from admission until 2 h afterwards, reaching less than 50%. Already a slightly compromised PT (≤ 70%) represented a significant factor (*p* < 0.05) for mortality at early and late time points, associated with a rate of more than 20%. In extremis, PT values of ≤ 25% were related to a mortality rate of more than 50% up to four hours after admission.

**Conclusion:**

There are early and significant differences in mortality according to PT values in polytrauma patients (despite resuscitation measures), urging for a fast correction of PT. Time-dependent and stratified referencing may help clinicians estimate the mortality risk and decide upon the extent of surgical care.

## Background

Polytrauma is associated with a high mortality rate. Patients suffering from polytrauma are commonly subject to coagulopathy [1–8], which can facilitate physical deterioration to the point of a lethal outcome [5–8].

Prothrombin time (PT) is commonly used to assess coagulative capacity. Several studies have confirmed its prognostic role for mortality in polytrauma patients [8–15], although results are confounded by injury severity [7, 9, 16–18], complicating the identification of cut-off values [19, 20]. Besides, most studies rely on PT values at admission [8–11], inhibiting re-assessment in the further course.

In the therapeutic sense, surgical interventions are regularly and urgently necessary to enable physiologic stabilization and return to function. If not performed as an urgent survival procedure, a surgery is generally intended as “definitive” treatment. It can, however, be forbidden in the first place by a compromised body function, as it would cause or at least contribute to the development of adverse events (AE), e.g. Systemic Inflammatory Response Syndrome (SIRS), sepsis, or even death [18, 21–30], possibly by an immunological response to trauma *(First Hit) *[13, 31, 32]. Consequently, only a “damage-control” surgery, e.g. external fixation, is performed to avoid an overshooting surgical load *(Second Hit) *[18, 28, 33], which will then require “definitive” treatment later on.

Polytrauma patients often suffer multiple surgery-indicating pathologies, which multiplies the aforementioned principle, making several interventions necessary. Regular re-assessment is required to decide upon the respective “window of opportunity” for each surgery to avoid AE, especially mortality.

As mortality in polytrauma remains high, there is a need for early mortality prediction [34]. Specifically, knowledge about time dependent cut-off values in PT values could help surgeons estimate the patients’ physiological state and thereby decide upon the extent of surgical treatment.

The aim was to analyze the time dependent role of PT in polytrauma patients related to mortality.

## Methods

### Ethical approval

Upon the development of the data base (Nr. StV: 1-2008) and the *Watson Trauma Pathway Explorer®* outcome tool (*BASEC 2021-00391*), ethical approval was granted by the ethical committee of the University Hospital Zurich. Research was performed according to the guidelines of good clinical practice and Helsinki, further by the TRIPOD statement regarding multivariable prediction model [35].

### Patient cohort

The study is based on an internal data base with ongoing admission. For this study, patients were considered since the beginning of recording in 1996 up until 2022.

Patients aged ≥ 16 years with an Injury Severity Score (ISS) ≥ 16 were included [36]. We excluded patients that died prior to admission or who were referred from other hospitals, as this would confound the timely aspect of measuring the parameters.

PT was measured at established time points (admission, 1, 2, 3, 4, 6, 8, 12, 24, and 48 h) [24, 26, 27, 29] after being admitted to our trauma bay at the University Hospital Zurich. Patients with missing admission parameters were also excluded.

Injury severity according to anatomic region was defined (Abbreviated Injury Score, AIS) [36] and the shock state [37] as well as resuscitation efforts were recorded.

### Measuring the prothrombin time

PT is reported as percentage activity of the reference reagence [%]. It was measured at our internal laboratory institute (*Institut für Klinische Chemie)*, using a standardized blood gas analyzer. All measurement were performed in the same manner for each time point. Measurements were ascribed to the nearest time point (as mentioned above).

### Statistical analysis

Analysis was performed using SPSS 29.0 (IBM SPSS Statistics 29). Values are reported according to scale level, defined as the mean with 95% confidence interval (CI) for numerical variables, median with interquartile range (IQR) for ordinal data and percentages for binary variables. PT is presented as median with IQR.

According to the status of survival, two groups were formed and compared by the Mann-Whitney-U-test, as there was no normal distribution and no equal variance.

Correlation analysis (Spearman correlation coefficient) was used to test the association between injury severity by injury region (AIS) and PT.

Binary logistic regression served for testing PT as an independent predictor for mortality, including cut-off values of ≤ 70% (which represents the cut-off for physiologic values in our institution), ≤ 50% and ≤ 25%. The injury severity according to ISS, New ISS (NISS) and Acute Physiology and Chronic Health Evaluation II (APACHE-II) score [7, 9, 16, 17, 29], further age [30] and sex [23, 30] were included during calculation to correct for confounding.

A closest top-left threshold method was used to calculate threshold values between the survivor and non-survivor group. For this, a receiver operating characteristic was created and the point closest to the top-left corner was identified, describing the maximum of combined sensitivity and specificity values.

Patients were divided into subgroups according to PT levels (> 70%, ≤ 70%, ≤ 50% and ≤ 25%) and the mortality rate was assessed for each subgroup at each time point. To analyze the association between subgroups and mortality for each time point, a Pearson Chi-Square test was used. Group sizes of 10 patients or less were not considered for analysis.

Significance was defined as *p* < 0.05.

## Results

From the identified cohort eligible for inclusion (3653 patients) [25–30], 2890 patients were included to account for missing PT values at admission.

The cohort represents a middle-aged group [mean 45.8 years (45.1-46.2 years)] of predominantly male patients (73.5%, *n* = 2124) suffering a mostly blunt trauma (91.8%, *n* = 2653). The mean accident-to-admission-interval was 65.2 min (63.9-66.5 min). Patients in the non-survivor group (30.5%, *n* = 882) had higher age [52.2 years (50.7-53.6 years) vs. 43.0 years (42.2-43.8 years); *p* < 0.001) and were subject to a greater injury severity according to the ISS [34 (IQR, 25-50) vs. 26 (20-34); *p* < 0.001], NISS [50 (41-66) vs. 34 (27-43); *p* < 0.001], and APACHE-II Score [22 (18-27) vs. 11 (6-18); *p* < 0.001]. Furthermore, lactate [4.2 mmol/L (3.9-4.4 mmol/L) vs. 2.5 mmol/L (2.5-2.6 mmol/L); *p* < 0.001] and heart rate [94 beats per minute (91-96 beats per minute) vs. 90 beats per minute (89-91 beats per minute); *p* < 0.001] were elevated.

Patients in the non-survivor group presented with a lower Glasgow Coma Score (GCS) [3 (3-3) vs. 13 (3-15); *p* < 0.001], body temperature [34.8 °C (34.6-35.0 °C) vs. 35.7 °C (35.6-35.8 °C); *p* < 0.001], systolic blood pressure [122.4 mmHg (119.4-125.5 mmHg) vs. 132.4 mmHg (131.2-13.6 mmHg); *p* = 0.022], hemoglobin [10.4 g/dL (10.0-10.7 g/dL) vs. 11.7 g/dL (11.5-11.8 g/dL); *p* < 0.001], pH [7.26 (7.25-7.27) vs. 7.33 (7.33-7.34); *p* < 0.001] and BE [−6.29 mmol/L (−6.80- −5.79 mmol/L) vs. −2.99 mmol/L (−3.19- −2.78 mmol/L); *p* < 0.001].

A lower body temperature showed a correlation with a lower PT at admission (*r* = 0.313; *p* < 0.001) and, at this admission point, represented a significant factor associated with it (*p* = 0.042) under correction for injury severity (ISS, NISS, APACHE-II Score), age and sex.

The number of values missing at each time (relative to total cohort) were: 0 at admission (0%), 2611 at 1 h (90.3%), 2636 at 2 h (91.2%), 2574 at 3 h (89.0%), 2370 at 4 h (82.0%), 2303 at 6 h (79.7%), 2359 at 8 h (81.6%), 1900 at 12 h (65.7%), 1064 at 24 h (36.8%), 1423 at 48 h (49.2%).

The injury profile according to anatomic region and severity is displayed in Table 1. The survivor group had lower injury severity in the head region and marginally higher injury severity in all other regions, except for comparable results for the pelvis region. Similarly, survivors had a lower rate of severe injuries to the head, chest and integument, but higher rates for the face, abdomen, pelvis, spine and extremities.

**Table 1:** Injury profile according to anatomic region and severity

Anatomic region	Survivor group	Median (IQR)	% AIS ≥3 (n)	Non- Survivor group (n)	Median (IQR)	% AIS ≥3 (n)	*p*-value (Median comparison)	*p*-value (Frequency comparison according to AIS ≥3)
Head		3 (0-4)	53.1 (1412)		5 (4-5)	85.8 (837)	**<0.001**	**<0.001**
Face		0 (0-1)	21.0 (552)		0 (0-0)	1.2 (69)	**<0.001**	**<0.001**
Chest		1 (0-3)	41.1 (1090)		0 (0-3)	42.5 (410)	**0.045**	**<0.001**
Abdomen		0 (0-2)	22.1 (580)		0 (0-0)	20.4 (196)	**<0.001**	**<0.001**
Pelvis		0 (0-0)	14.3 (375)		0 (0-0)	13.5 (129)	0.981	**0.003**
Spine		0 (0-2)	17.1 (447)		0 (0-0)	11.2 (107)	**<0.001**	**<0.001**
Extremities		2 (0-3)	28.7 (760)		0 (0-2)	19.5 (186)	**<0.001**	**<0.001**
Integument		0 (0-1)	2.0 (51)		0 (0-1)	3.3 (31)	**<0.001**	**<0.001**

*AIS *Abbreviated Injury Score, *IQR *Interquartile Range

There were 399 patients (10.9%) at admission showing a shock index of ≥ 1, of which 75 (2.1%) demonstrated a severy shock state with an index of ≥ 1.5.

Resuscitation efforts by transfusions and factor corrections are displayed in Table 2. Here, red blood cell concentrate admission was higher for the non-survivor group in frequency from 1 h to 48 h after admission and in mean at 1, 2, 3 and 48 h thereafter. Differences for fresh frozen plasma existed at 1 h to 3 h after admission, respectively. Fibrinogen was applied more often at 2, 6, 8, 24 and 48 h while having a larger dose at 1, 2, 8, 12, 24, and 48 h after admission. There were no group differences for the frequency or mean of prothrombin complex concentrate administration.

**Table 2:** Resuscitation efforts by transfusions and factor corrections according to frequency and mean values

Parameter	Survivor group	% of patients receiving (n)	Mean (95% CI)	Non-Survivor group	% of patients receiving (n)	Mean (95% CI)	*p*-value (Frequency comparison)	*p*-value (Mean comparison)
RBCC at admission		1.3 (25)	0.04 (0.01-0.07)		1.3 (11)	0.04 (0-0.08)	0.400	0.961
RBCC 1 h		7.0 (135)	0.30 (0.23-0.37)		15.7 (132)	0.95 (0.74-1.17)	**<0.001**	**<0.001**
RBCC 2 h		15.7 (304)	0.84 (0.71-0.97)		21.1 (178)	1.76 (1.41-2.11)	**<0.001**	**<0.001**
RBCC 3 h		16.2 (316)	1.33 (1.14-1.51)		23.4 (195)	2.42 (1.95-2.89)	**<0.001**	**0.03**
RBCC 4 h		25.7 (499)	1.68 (1.46-1.90)		25.6 (213)	2.78 (2.25-3.31)	**<0.001**	0.127
RBCC 6 h		28.1 (548)	2.11 (1.85-2.37)		26.6 (222)	3.27 (2.65-3.89)	**<0.001**	0.729
RBCC 8 h		30.1 (586)	2.39 (2.11-2.68)		25.8 (212)	3.48 (2.82-4.13)	**<0.001**	0.601
RBCC 12 h		34.8 (678)	2.92 (2.60-3.24)		31.8 (261)	4.10 (3.36-4.84)	**0.004**	0.667
RBCC 24 h		38.5 (752)	3.42 (3.05-3.78)		21.4 (176)	4.42 (3.63-5.22)	**0.002**	0.216
RBCC 48 h		41.6 (818)	3.95 (3.54-4.35)		35.0 (286)	4.71 (3.88-5.53)	**<0.001**	**0.034**
FFP at admission		0.5 (9)	0.01 (0-0.02)		0.5 (4)	0.02 (0-0.03	0.520	0.966
FFP 1 h		1.4 (26)	0.04 (0.02-0.05)		5.5 (46)	0.20 (0.13-0.28)	**<0.001**	**<0.001**
FFP 2 h		7.6 (146)	0.37 (0.29-0.44)		12.8 (106)	0.76 (0.57-0.95)	**0.002**	**<0.001**
FFP 3 h		12.2 (233)	0.77 (0.64-0.90)		15.4 (127)	1.32 (1.02-1.63)	**<0.001**	**0.01**
FFP 4 h		15.3 (293)	1.11 (0.95-1.28)		17.2 (141)	1.65 (1.27-2.03)	0.071	0.141
FFP 6 h		17.6 (337)	1.53 (1.32-1.75)		18.9 (154)	2.06 (1.60-2.51)	0.343	0.258
FFP 8 h		20.1 (385)	1.79 (1.55-2.03)		19.9 (162)	2.29 (1.79-2.79)	0.120	0.846
FFP 12 h		24.0 (459)	2.31 (2.03-2.60)		23.2 (188)	2.80 (2.21-3.39)	0.625	0.831
FFP 24 h		25.5 (488)	2.78 (2.44-3.13)		23.3 (189)	3.12 (2.45-3.79)	0.460	0.328
FFP 48 h		26.5 (507)	3.25 (2.85-3.64)		23.2 (187)	3.33 (2.61-4.06)	0.387	0.100
Fibrinogen at admission		0.3 (5)	0.01 (0-0.01)		0.4 (3)	0.01 (0-0.02)	0.310	0.645
Fibrinogen 1 h		3.8 (72)	0.10 (0.07-0.13)		5.6 (46)	0.13 (0.06-0.19)	0.126	0.031
Fibrinogen 2 h		7.6 (146)	0.24 (0.19-0.29)		10.2 (84)	0.35 (0.23-0.47)	**0.019**	**0.02**
Fibrinogen 3 h		10.8 (207)	0.38 (0.31-0.44)		11.9 (97)	0.60 (0.31-0.89)	0.079	0.326
Fibrinogen 4 h		13.0 (249)	0.50 (0.42-0.58)		12.8 (103)	0.73 (0.41-1.05)	0.064	0.995
Fibrinogen 6 h		16.5 (317)	0.65 (0.55-0.74		14.0 (113)	1.09 (0.47-1.71)	**0.004**	0.161
Fibrinogen 8 h		18.8 (360)	0.80 (0.65-0.95)		14.4 (115)	1.14 (0.52-1.77)	**0.003**	**0.014**
Fibrinogen 12 h		20.3 (387)	0.87 (0.71-1.02)		16.0 (127)	1.20 (0.58-1.83)	0.055	**0.019**
Fibrinogen 24 h		21.1 (403)	0.98 (0.79-1.17)		16.1 (128)	1.38 (0.52-2.23)	**0.004**	**0.007**
Fibrinogen 48 h		21.2 (404)	1.00 (0.81-1.19)		15.5 (122)	1.38 (0.52-2.23)	**0.015**	**0.001**
PCC at admission		0.2 (4)	4.09 (0.75-8.93)		0.3 (3)	0	0.287	0.484
PCC 1 h		0.8 (16)	11.83 (4.54-19.12)		0.9 (8)	9.80 (0.02-19.81)	0.287	0.774
PCC 2 h		14.7 (30)	19.15 (10.73-27.58)		2.3 (21)	20.59 (7.88-33.30)	0.279	0.096
PCC 3 h		2.5 (52)	33.53 (21.49-45.57)		2.8 (25)	29.74 (12.58-46.89)	0.641	0.700
PCC 4 h		2.9 (60)	40.30 (26.76-53.84)		3.6 (32)	41.50 (19.75-63.25)	0.320	0.354
PCC 6 h		3.6 (74)	51.33 (35.88-66.77)		4.0 (36)	54.77 (28.33-81.21)	0.238	0.582
PCC 8 h		3.8 (78)	55.64 (39.55-71.72)		4.4 (39)	65.23 (35.80-94.65)	0.634	0.467
PCC 12 h		4.2 (85)	62.03 (44.97-79.09)		4.4 (39)	70.46 (40.32-100.60)	0.420	0.749
PCC 24 h		4.4 (89)	68.53 (50-87.06)		4.4 (39)	75.69 (42.68-108.69)	0.593	0.938
PCC 48 h		4.4 (90)	71.29 (52.19-90.40)		4.1 (36)	85.62 (47.01-124.23)	0.627	0.705

*CI *Confidence interval, *FFP *Fresh frozen plasma [number of packed units]; Fibrinogen [g/L], *PCC* [international units] Prothrombin complex concentrate, *RBCC *Red blood cell concentrate [number of packed units]

Correlation analysis revealed significant associations between increasing injury severity by region and a lower PT (Table 3), decreasing timewise from extremities/pelvis (over the first 24 h), thorax (12 h) and abdomen (8 h). Inconsistent differences existed for the spine and integument as well as the head region. The head region even showed a positive correlation at 4 h and 6 h. No association was observed for the face region.

**Table 3:** Correlation between injury region and prothrombin time according to the predefined time point. Data is reported as Spearman correlation coefficient; with the p-value in brackets

Parameter	Admission	1 h	2 h	3 h	4 h	6 h	8 h	12 h	24 h	48 h
Head	**-0.047 ** (0.021)	-0.034 (0.596)	0.121 (0.075)	0.048 (0.427)	**0.204** **(<0.001)**	**0.091** **(0.039)**	0.018 (0.699)	-0.054 (0.122)	**-0.062** **(0.015)**	**-0.154** **(<0.001)**
Face	0.025 (0.228)	0.006 (0.930)	0.030 (0.659)	0.037 (0.537)	0.071 (0.136)	0.013 (0.764)	0.008 (0.865)	-0.015 (0.666)	0.005 (0.855)	0.004 (0.899)
Thorax	**-0.167** **(<0.001)**	**-0.249** **(<0.001)**	**-0.251** **(<0.001)**	**-0.313** **(<0.001)**	**-0.295** **(<0.001)**	**-0.097** **(0.029)**	**-0.174** **(<0.001)**	**-0.069** **(0.05)**	-0.039 (0.127)	0.031 (0.287)
Abdomen	**-0.160** **(<0.001)**	**-0.314** **(<0.001)**	**-0.370** **(<0.001)**	**-0.313** **(<0.001)**	**-0.267** **(<0.001)**	**-0.143** **(0.001)**	**-0.103** **(0.03)**	-0.062 (0.08)	-0.047 (0.067)	-0.018 (0.547)
Pelvis	**-0.187** **(<0.001)**	**-0.326** **(<0.001)**	**-0.306** **(<0.001)**	**-0.203** **(<0.001)**	**-0.185** **(<0.001)**	**-0.123** **(0.005)**	**-0.100** **(0.036)**	**-0.098** **(0.005)**	**-0.085** **(<0.001)**	-0.029 (0.313)
Spine	**-0.051** **(0.013**	-0.107 (0.098)	**-0.158** **(0.021)**	**-0.216** **(<0.001)**	**-0.173** **(<0.001)**	**-0.103** **(0.02)**	-0.052 (0.277)	-0.02 (0.570)	0.03 (0.238)	**0.061** **(0.036)**
Extremities	**-0.156** **(<0.001)**	**-0.305** **(<0.001)**	**-0.344** **(<0.001)**	**-0.296** **(<0.001)**	**-0.254** **(<0.001)**	**-0.146** **(<0.001)**	**-0.094** **(0.049)**	**-0.124** **(<0.001)**	**-0.100** **(<0.001)**	-0.004 (0.886)
Integument	**-0.061** **0.003**	-0.046 (0.482)	**-0.175** **(0.01)**	**0.223** **(<0.001)**	**-0.177** **(<0.001)**	-0.073 (0.104)	**-0.097** **(0.042)**	**-0.077** **(0.03)**	-0.05 (0.051)	-0.007 (0.806)

### Differences in prothrombin time according to survivor status

**Fig. 1:**
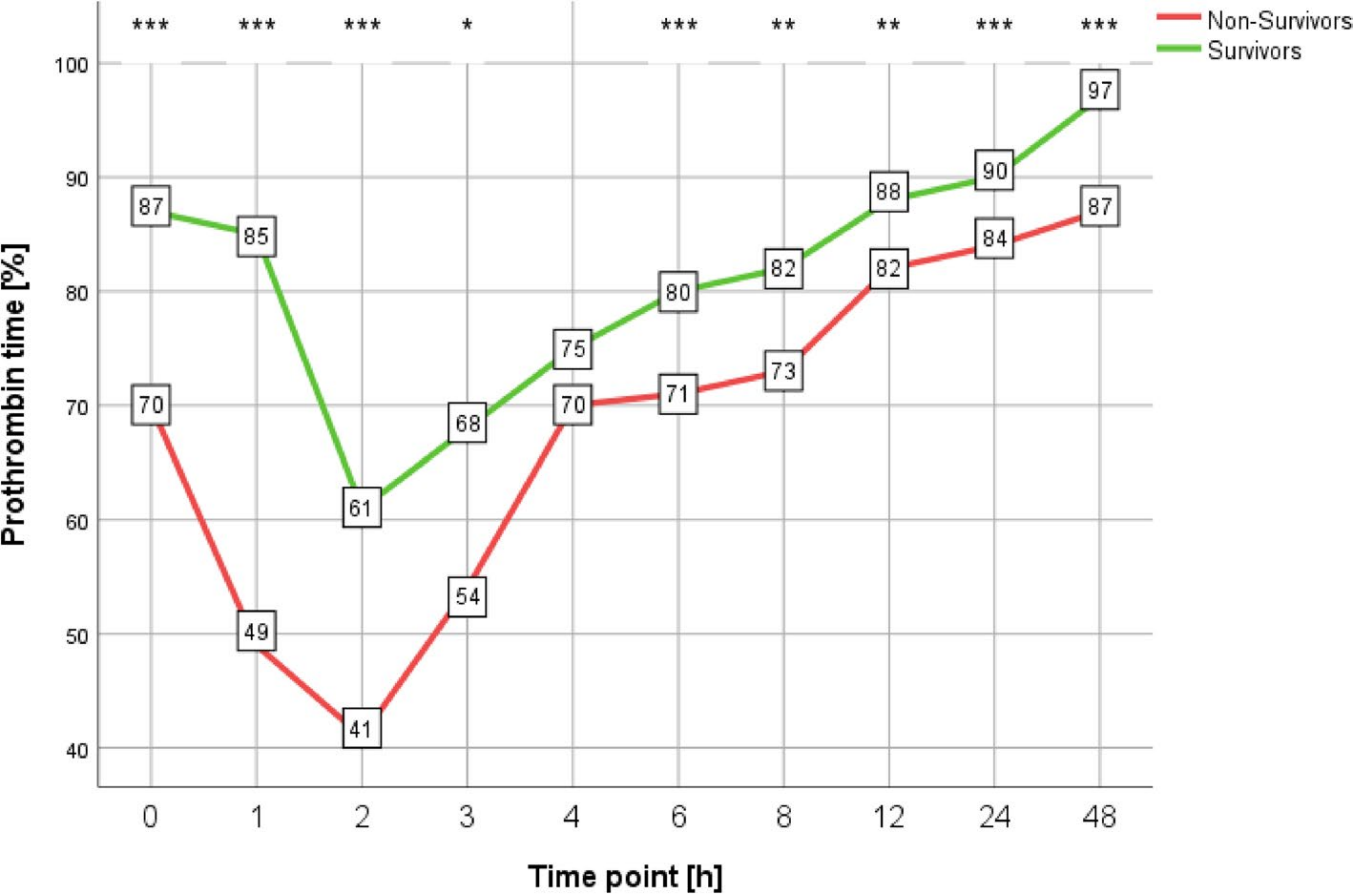
Differences in prothrombin time according to survivor status. *p < 0.05; **p ≤ 0.01; ***p ≤ 0.001

PT values in the non-survivor group were lower throughout the measuring period (Fig. 1). PT values declined from admission until 2 h afterwards, reaching less than 50% PT at 1 h and 2 h for the non-survivor group. They then recovered, independently of survivor status.

### Prothrombin time as an independent predictor for mortality

**Fig. 2:**
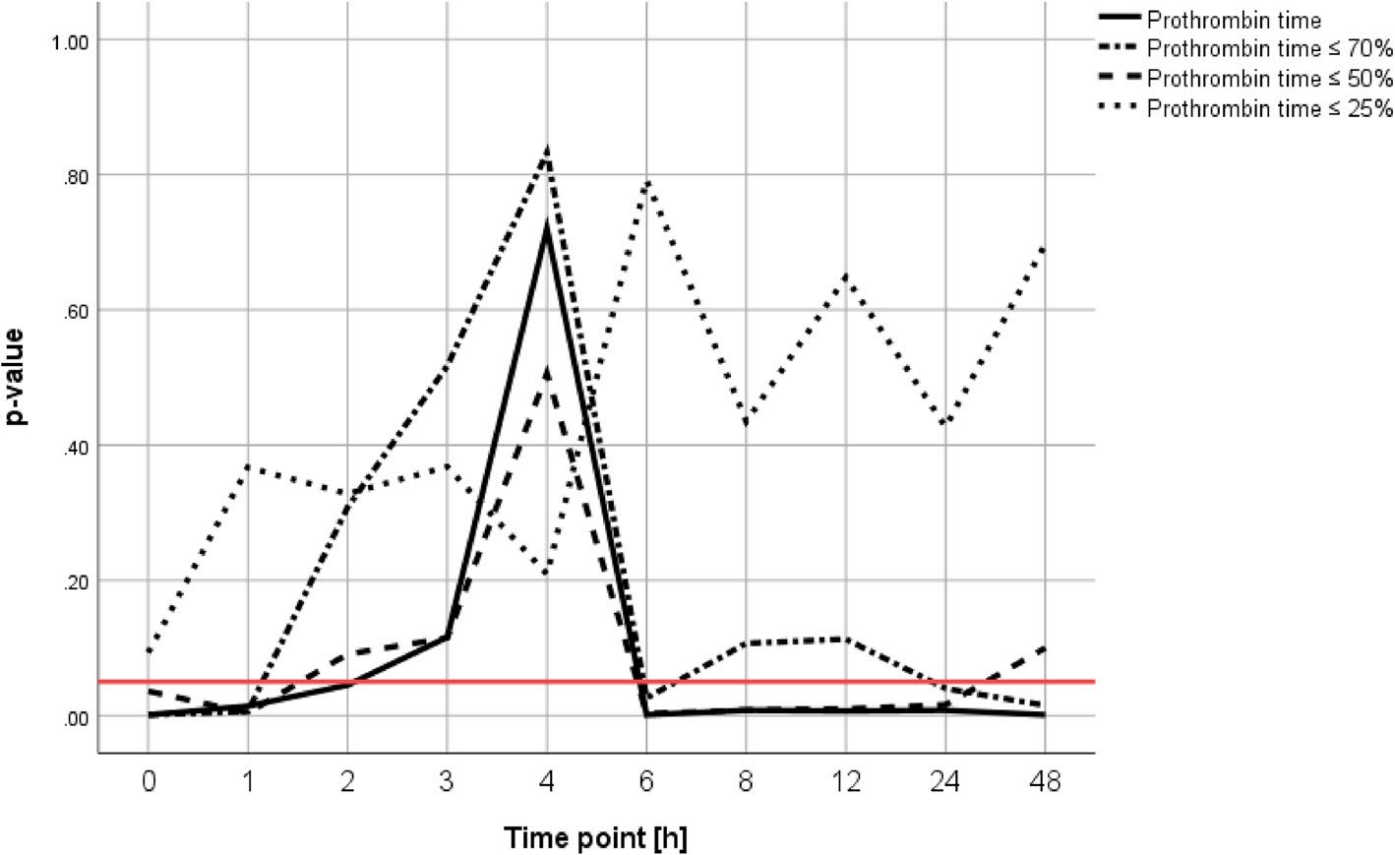
Prothrombin time as an independent predictor for mortality. Analysis was corrected for injury severity, age and sex. It includes cut-off values of ≤ 70%, ≤ 50% and ≤ 25%. The red horizontal line indicates the level of significance, being *p* = 0.05

After correcting for injury severity, age and sex, PT represented an independent predictor within the first two hours (admission: *p* < 0.001; 1 h: *p* = 0.014; 2 h: *p* = 0.045) and from 6 to 48 h (6 h: *p* < 0.001; 8 h: *p* = 0.008; 12 h: *p* = 0.007; 24 h: *p* = 0.008; 48 h: *p* = 0.001) (Fig. 2). Using a cut-off value of ≤ 70%, prediction was significant at admission (*p* < 0.001); 1 h (*p* = 0.006); 6 h (*p* = 0.026); 24 h (*p* = 0.040) and 48 h (*p* = 0.015). Similarly, a cut-off value of ≤ 50% was significant at admission (*p* = 0.036); 1 h (*p* = 0.004); and from 6 to 24 h (6 h: *p* = 0.003; 8 h: *p* = 0.008; 12 h: *p* = 0.010; 24 h: *p* = 0.016). A cut-off value of ≤ 25% only tended to be significant at admission (*p* = 0.093).

### Threshold values in prothrombin time according to survivor status

**Fig. 3:**
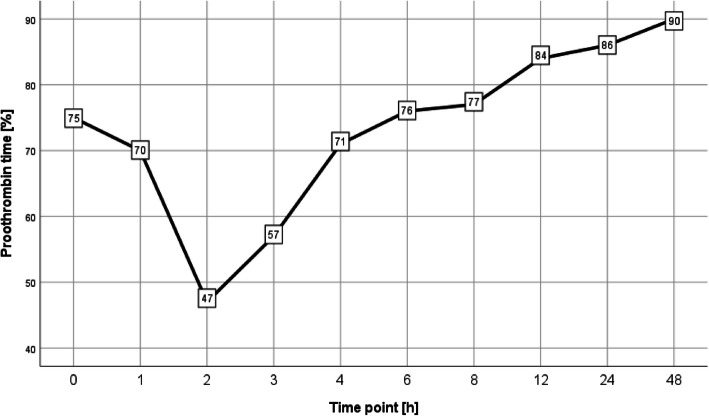
Threshold values in prothrombin time according to survivor status

Similar to the above-mentioned group analysis, PT threshold values declined from admission until 2 h afterwards, reaching less than 50% at 2 h (Fig. 3). They recovered afterwards until the end of the observational period.

### Mortality rates according to stratified prothrombin time values

**Fig. 4:**
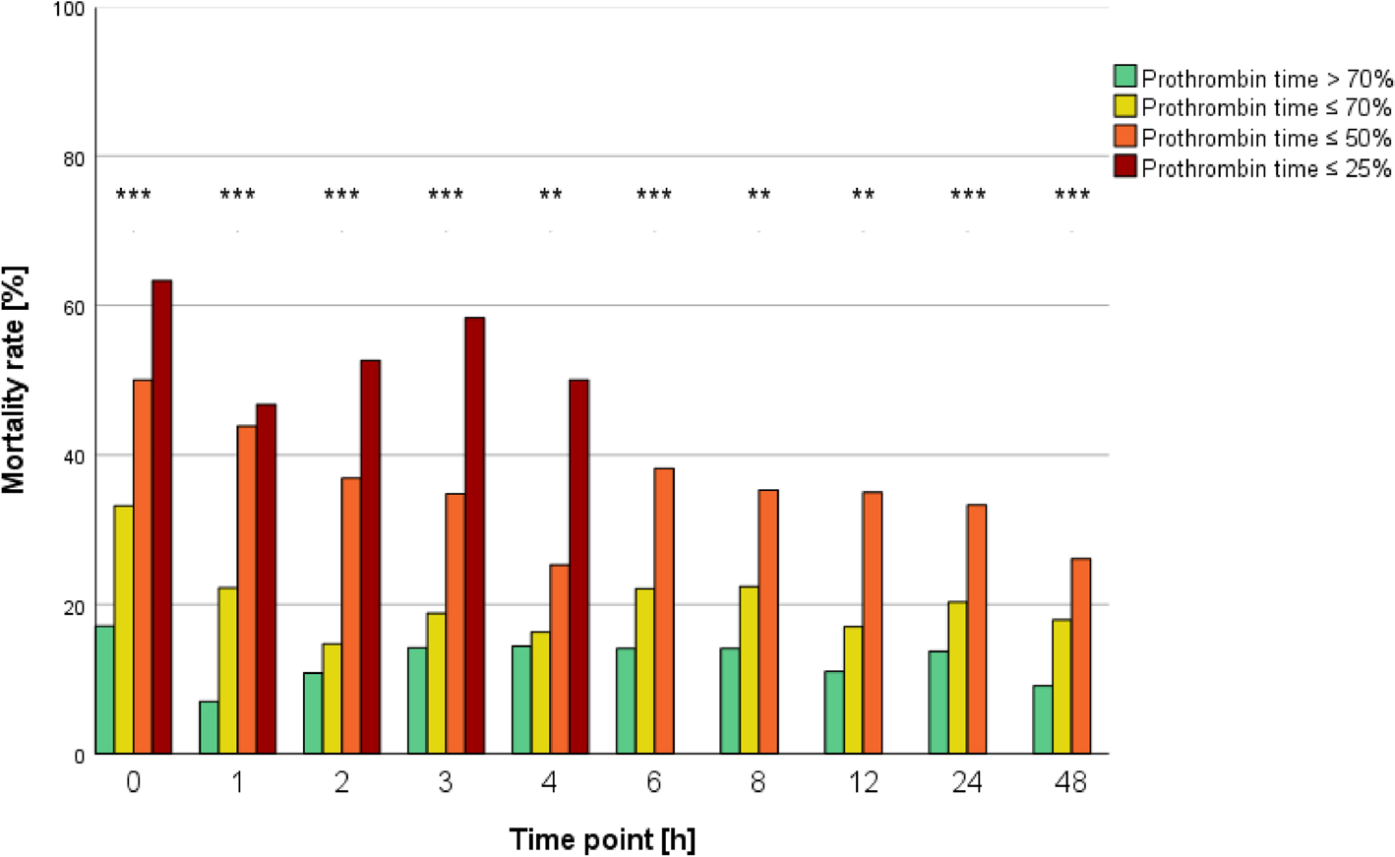
Mortality rates according to stratified prothrombin time values. No analysis or graphic representation was performed for cases with a prothrombin time of ≤ 25% after 4 h as each group was less than ten. *p < 0.05; **p ≤ 0.01; ***p ≤ 0.001

For each time point, increasing coagulopathy according to PT was associated with a higher mortality rate (Fig. 4).

Mortality rates were generally stable for each group according to PT. Since group sizes for a PT of ≤ 25% were less than 10 patients from 6 h onwards, no analysis or graphic representation was performed for these aspects.

## Discussion

This study confirms the early predictive role of PT for mortality in polytrauma patients, as indicated by foregoing studies [8–15], specifically by a similar study group with an admission PT of 84 for survivors at an ISS of 29 [7]. Injury severity is a fundamental confounding factor [7, 9, 16–18, 28–30], but has been corrected for statistically.

Regarding injury pattern, a head-centered injury pattern was confirmed in its relation to mortality [38, 39]. Looking at the correlation of injury severity by anatomic region and PT (based on the concept of coagulopathy caused by traumatic bleeding [40]), the thorax and abdomen are crucial in consideration and are especially relevant if the central (aortic) vessels and the (left) heart are affected [41]. The relevance is also true for the extremity and pelvic region, echoing previous reports stressing their relevance in trauma [41]. Heterogenic results for the head region are congruent with the notion that such injuries provoke a unique type of coagulopathy and modify the process and outcome of it substantially [40].

PT should directly be considered at admission, as it differed virtually permanently since then, in light of a higher injury severity for non-survivors. It represents a known identity and has correlation with immunology and coagulation pathways [40, 42, 43]. Despite efforts of resuscitation (pharmaceutic and/or surgical) a substantial coagulopathy according to PT takes place [7].

Regarding specific cut-off values, it appears that a PT value of approximately 50% at 2 h can be used as an early differentiation between survivors and non-survivors.

Even slightly abnormal PT values were associated with mortality in the later course, of which surgeons should be aware of and perform a regular re-assessment.

Time-dependent risk-stratification showed a linear correlation between the degree of coagulopathy (PT) and mortality, suggesting that roughly every third patient with a PT ≤ 50% and more than half of the patients with a PT ≤ 25% die after polytrauma. This reaffirms the aspect of injury severity [7, 9, 16–18, 28–30]. The finding with less than an ambidextrous number of survivors ≤ 25% at later time points is seen as reason for the missing significance (type II-error) at these points. These consequently excluded values of patients with a PT ≤ 25% as early as 6 h due to few cases after high mortality [7, 15] speak for themselves.

Several limitations must be mentioned: Patient medical history (especially hemostasis altering by diseases/medication) was not considered. The cohort was not analysed according to injury pattern, which might be associated with anatomic regions more prone to bleeding. PT values are inherently confounded by resuscitation (dilution) and transfusion (functional capacity), and changes in PT could be more important than numbers at different time points. Resuscitation protocols (including the extent of surgical treatment) were performed according to current standards over the time frame of 1996 to 2022.

In this regard, first studies reporting an association between coagulopathy and mortality in polytrauma patients were described in 2003 [11, 44], with an increasing number since then, expanding the knowledge on coagulopathy in polytrauma patients. The increasing insights led to changes in transfusion protocols, reducing trauma-induced hemorrhage and mortality [45]. Yet, correction of hemorrhage remains in the acute phase remains challenging and subject to further research [46–48]. The measuring period is rather short at 48 h, as it was reserved to intensive care units. Despite the large study size, there were only few cases with a PT of ≤ 25% (as a consequence of high mortality), which inhibited group analysis in the later time course.

Ultimately, this study was able to present a comprehensive profile of PT in polytrauma patients, urging for a fast correction of PT (in consideration of hypothermia [1, 4]). PT can aid in decision-making by providing a reference. Clinicians can estimate the risk, which dictates the possible extent of pharmaceutical or surgical care (damage-control, early total care) to provide maximal treatment effect while minimizing AE. Thereby, patient outcomes after polytrauma shall be improved.

## Conclusions

There are early and significant differences in mortality according to PT values (despite resuscitation measures), urging for a fast correction of PT. Time-dependent and stratified referencing may help clinicians estimate the mortality risk and decide upon the extent of surgical care.

## Data Availability

No datasets were generated or analysed during the current study.

## Abbreviations

AE: Adverse Events
AIS: Abbreviated Injury Score
APACHE-II: Acute Physiology and Chronic Health Evaluation II
CI: Confidence Interval
FFP: Fresh Frozen Plasma
GCS: Glasgow Coma Scale
IQR: Interquartile Range
ISS: Injury Severity Score
NISS: New ISS
PCC: Prothrombin Complex Concentrate
PT: Prothrombin time
RBCC: Red Blood Cell Concentrate
SIRS: Systemic Inflammatory Response Syndrome
